# rHuEPO Hyporesponsiveness and Related High Dosages Are Associated with Hyperviscosity in Maintenance Hemodialysis Patients

**DOI:** 10.1155/2013/792698

**Published:** 2013-09-30

**Authors:** Mehtap Erkmen Uyar, Selami Kocak Toprak, Hatice Saglam, Emre Tutal, Meltem Bay, Osman Ilhan, Zeynep Bal, Siren Sezer

**Affiliations:** ^1^Department of Nephrology, Baskent University Medical School, 06490 Ankara, Turkey; ^2^Department of Hematology, Baskent University Medical School, 06490 Ankara, Turkey; ^3^Department of Internal Medicine, Baskent University Medical School, 06490 Ankara, Turkey; ^4^Department of Hematology, Ankara University Medical School, 06490 Ankara, Turkey

## Abstract

*Objective*. Increased viscosity may increase the risk of thrombosis or thromboembolic events. Recombinant human erythropoietin (rHuEPO) is the key stone treatment in anemic ESRD patients with the thrombotic limiting side effect. We evaluated the influence of clinical and laboratory findings on plasma viscosity in MHD patients in the present study. *Method*. After applying exclusion criteria 84 eligible MHD patients were included (30 female, age: 54.7 ± 13.7 years). *Results*. Patients with high viscosity had longer MHD history, calcium × phosphorus product, and higher rHuEPO requirement (356.4 versus 204.2 U/kg/week, *P*: 0.006). rHuEPO hyporesponsiveness was also more common in hyperviscosity group. According to HD duration, no rHuEPO group had the longest and the low rHuEPO dosage group had the shortest duration. Despite similar Hb levels, 68% of patients in high rHuEPO dosage group; and 38.7% of patients in low rHuEPO dosage group had higher plasma viscosity (*P*: 0.001). Patients with hyperviscosity had higher rHuEPO/Hb levels (*P*: 0.021). Binary logistic regression analyses revealed that rHuEPO hyporesponsiveness was the major determinant of hyperviscosity. *Conclusion*. We suggest that the hyperviscous state of the hemodialysis patients may arise from the inflammatory situation of long term HD, the calcium-phosphorus mineral abnormalities, rHuEPO hyporesponsiveness, and related high dosage requirements.

## 1. Introduction 

The resistance of blood against blood flow is called plasma viscosity. Blood is a complex body fluid, so not only body temperature but also components of blood like hematocrit and plasma and rheological characteristics like the deformability of erythrocytes all affect plasma viscosity [1]. Plasma viscosity is influenced by diseases with altered plasma protein composition, determined by various macromolecules, for example, fibrinogen, immunoglobulin, and lipoproteins [2, 3]. An elevated viscosity significantly increases the risk of inflammatory diseases; the strong positive correlation between plasma viscosity and fibrinogen has been reported in several studies [2, 3]. 

Chronic kidney disease (stages 4 and 5) (CKD) is also associated with alterations of coagulation that favor a hypercoagulable or prothrombotic state [4, 5] and thus an increased thrombotic risk that may contribute to an increase in cardiovascular morbidity and mortality [6]. In the uremic state, mechanisms including hypertension, hyperhomocysteinemia, dyslipidemia with high lipoprotein (a) (Lp(a)) levels, elevation of hemostatic derived cardiovascular risk factors (fibrinogen and proconvertin) [6], and amplification of the inflammatory cascade at the endothelial cell (growth factors, cytokines, and adhesion molecules) [7] are activated [8]. The characteristic of blood flow is closely related to the blood flow circumstances with all the factors above as seen in atherosclerotic diseases [9].

Elevated plasma viscosity results in greater flow resistance and a high incidence of circulatory complications [10]. Increased blood and plasma viscosity has been described in patients with coronary and peripheral arterial disease. An increase in factor VII plasma levels, hematocrit, and platelet function [11–13] and a decrease in anticoagulant protein S, C, and antithrombin III [14, 15] have been described in ESRD. Increased viscosity may increase the risk of thrombosis or thromboembolic events [16].

In ESRD patients the limiting side effect of recombinant human erythropoietin (rHuEPO) is the thrombotic side effect. Several reports have associated long-term rHuEPO treatment with thrombotic complications of arteriovenous fistulae, convulsion and cerebrovascular disease, hyperviscosity, and hypertension [17–21]. In patients undergoing dialysis, the risk of death has been shown to be inversely associated with a good hematopoietic response to rHuEPO [22]. It has specifically been shown in the TREAT cohort that poor hemoglobin response to ESA treatment is associated with poor outcomes [23]. The patients with the poorest initial response received the highest average doses of ESAs and had the highest event rates [24]. Also the weak association between a poor initial response and the C-reactive protein level also suggests that inflammatory factors may contribute to a poor initial response [25]. Normal hemoglobin levels in patients without rHuEPO usage had no influence on mortality [25].

In the light of all these data, we planned to evaluate the influence of clinical and laboratory findings including iron parameters, hemoglobin, albumin, CRP, parathyroid hormone, and monthly rHuEPO requirements on plasma viscosity in maintenance hemodialysis (MHD) patients in this present study.

## 2. Patients and Methods

258 ESRD patients receiving MHD in our hemodialysis unit for at least 12 months were prospectively analyzed for 12 months. Patients who had active infection, iron deficiency (ferritin levels < 200 mg/dL or transferrin saturation < 20%), malignancy, severe clinical malnutrition, receiving antiaggregant, or anticoagulant therapy (except intradialytic heparinization) were excluded. After applying exclusion criteria 84 eligible patients were included (30 female, age: 54.7 ± 13.7 years). Informed consent was obtained in each patient. Plasma viscosity was studied in a fasting blood sample which was sampled just before a clinically stable HD session. Plasma viscosities of all subjects were measured at 37°C in a Brookfield DV-II + Clone Plate Viscometer [Brookfield, Stoughton, MA, USA]. Data including iron parameters and treatment dosage, hemoglobin, albumin, CRP, calcium, phosphorus, parathyroid hormone, and monthly rHuEPO requirement were collected from patient charts and a mean value of the 12 months follow-up period was recorded as the final data. Patients' thromboembolic events like myocardial infarction, cerebrovascular disease, deep venous thrombosis and pulmonary thromboembolism, and arteriovenous fistula complications (like thrombus, emboli, or clotting) were analyzed and recorded in the one-year followup. 

Study group was divided into two equal sized groups as high (*n*: 42) and low viscosity groups (*n*: 42) for statistical analysis. Then these two groups were compared with each other in means of demographical and biochemical characteristics. rHuEPO hyporesponsiveness was defined as rHuEPO requirements >150 U/kg/week to achieve a target hemoglobin level of 11-12 g/dL. We classified patients receiving rHuEPO (>150 U/kg/week) as hyporesponsive (*n*: 40), low dose rHuEPO (75–300 U/kg/week) (*n*: 23), and no rHuEPO (*n*: 21) groups. 

### 2.1. Statistical Analysis

Statistical analyses were performed by using SPSS software (Statistical Package for the Social Sciences, version 11.0, SSPS Inc., Chicago, IL, USA). Normality of data was analyzed by using a Kolmogorov-Smirnov test. All numerical variables with normal distribution were expressed as the means ± standard deviations (SD), while variables with skew distribution were expressed as medians and interquartile range (IR). Categorical variables were expressed as percentages and compared by chi-square test. Normally distributed numeric variables were analyzed by independent samples *t*- or one-way ANOVA (post hoc Tukey) tests according to distribution normality. Skew distributed numeric variables were compared using the Mann-Whitney *U* and Kruskal-Wallis tests according to distribution normality. Spearman and Pearson Correlation tests were used for correlation analyses. A *P* value <0.05 was considered as statistically significant.

## 3. Results

**Figure 1:**
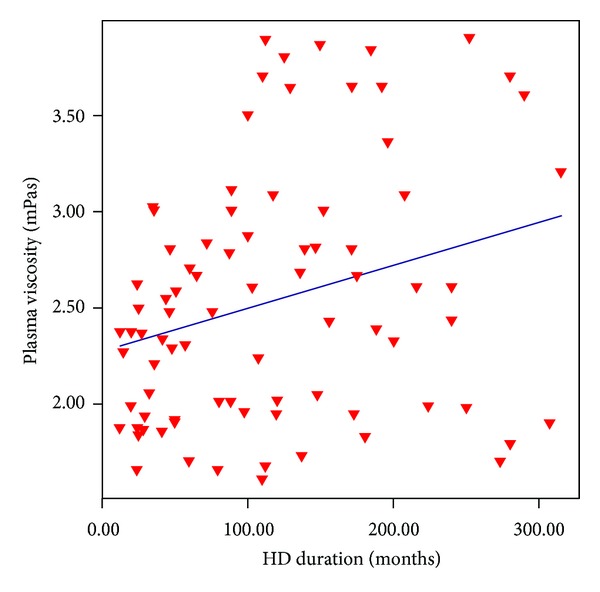
Plasma viscosity was positively correlated with patients' hemodialysis duration (*r*: 0.287, *P*: 0.008).

Demographic and biochemical characteristics of study groups are summarized in Table 1. Plasma viscosity of whole study group was 2.52 ± 0.65 (range 1.6–3.9) mPas. Plasma viscosity was positively correlated with duration of MHD (*r*: 0.287, *P*: 0.008, Figure 1). Comparison of high and low viscosity groups revealed that patients with high viscosity had longer MHD history (133.2 ± 77.1 versus 97.5 ± 83.1 months, *P*: 0.044), higher calcium (9.51 ± 0.61 versus 9.13 ± 0.65 mg/dL, *P*: 0.009), phosphorus (5.18 ± 0.8 versus 4.71 ± 1.09 mg/dL, *P*: 0.035), calcium × phosphorus product (49.37 ± 9.39 versus 43.45 ± 11.4, *P*: 0.011), and higher rHuEPO requirement (356.4 (295.7) versus 204.2 (350.8) U/kg/week, *P*: 0.006, Table 2). rHuEPO hyporesponsiveness was also more common in hyperviscosity group (28/42, 66.7% versus 13/42, 31%, *P*: 0.004). those of Patients with hyperviscosity had higher rHuEPO/Hb levels (*P*: 0.021). In hyperviscosity group the rHuEPO requirements of patients were higher (28/42, 66.7% versus 13/42, 31%, *P*: 0.004) than patients having lower viscosity. 

According to the one-year follow-up data of the patients, thromboembolic complications of arteriovenous fistula were significantly higher in patients with hyperviscosity in than patients with lower viscosity (54.8% versus 36.3%, *P*: 0.023). Other thromboembolic events like myocardial infarction, cerebrovascular disease, deep venous thrombosis, pulmonary thromboembolism, and mortality rates were similar between the two groups (Table 3). Acute myocardial infarction, pulmonary thromboembolism, and mortality rates were also not significant but higher in hyperviscosity group (Table 3).

Plasma viscosity of patients with no rHuEPO group (*n* = 21), low rHuEPO dosage group (*n* = 31), and high rHuEPO dosage group (*n* = 32), was 2.5 ± 0.6, 2.4 ± 0.7 and 2.6 ± 0.5, respectively (*P* > 0.05). The clinical and biochemical characteristics of rHuEPO user and nonuser patients are summarized in Table 4. In the three groups, there was no statistically significant difference in plasma viscosity of patients between different Hb levels (*P* > 0.05). Despite similar Hb levels (11-12 g/dL), 68% of patients in high rHuEPO dosage group and 38.7% of patients in low rHuEPO dosage group had higher plasma viscosity (*P*: 0.001). According to HD duration, no rHuEPO group had the longest and the low rHuEPO dosage group had the shortest HD duration. 

Binary logistic regression analyses revealed that rHuEPO hyporesponsiveness was the major determinant of hyperviscosity (*P*: 0.001).

## 4. Discussion

Hyperviscosity has effects leading to atherosclerosis, and its negative impact on atherosclerosis was found to be more intense than that of the traditional risk factors [26, 27]. Increased viscosity also has negative impact on vascular structure. Yarnell et al. found that in a population of 4860 men, death, acute myocardial infraction, and urgent cardiovascular surgery requirement were significantly higher in hyperviscosity group than in patients with lower blood viscosity [28]. On the other hand traditional cardiovascular risk factors like hypertension, obesity, smoking, high LDL-cholesterol levels and diabetes are also known to cause hyperviscosity [29–31]. Therefore, the interaction between blood viscosity and cardiovascular risk factors is complex but undeniable [32].

Survival of ESRD patients is significantly lower than that of normal population. Factors associated with increased mortality in ESRD were extensively studied and some significant factors like chronic inflammation, malnutrition, hyperphosphatemia, increased calcium × phosphorus product levels, and severe anemia are already defined as factors associated with all-cause and atherosclerosis related mortality [33–38]. In a study by Suzuki et al., the severity of atherosclerosis in maintenance hemodialysis patients was dependent on age and HD, gender, dyslipidemia, smoking, HD therapy, and HD duration [39].

In this study, we found higher plasma viscosity levels in ESRD patients undergoing MHD than that in normal population. We also found longer MHD duration, higher calcium, phosphorus, calcium × phosphorus product values, and higher dose rHuEPO requirement in patients with high viscosity. rHuEPO hyporesponsiveness was also more common in hyperviscosity group as for similar Hb levels higher dosage rHuEPO were required in this group. Longer HD duration and high dosage rHuEPO usage due to rHuEPO hyporesponsiveness were the major determinants of hyperviscosity.

In chronic kidney disease, serum calcium and especially phosphorus levels have been associated with vascular calcification and atherosclerosis [40]. Studies have shown a correlation between elevated phosphorus levels in dialysis and mortality [41]. Phosphorus has been shown to be an independent risk factor for cardiovascular disease [42], including increased intima media thickness [43–45], vessel stiffness [46, 47], and left ventricular hypertrophy [44]; PTH per se may contribute to vascular injury via mechanisms other than its effect on calcium-phosphorus homeostasis [48]. In previous studies, a strong correlation between higher rates of vascular calcification, cardiovascular mortality, and malnutrition and endothelial dysfunction and inflammation states were found in dialysis patients [49–51]. Serum fetuin-A levels, as both a calcification inhibitor protein and a negative acute-phase reactant, are significantly lower in dialysis patients and results of these studies suggest a link between inflammation and atherosclerosis in these patients [49–51]. In this present study, patients with high viscosity levels were found to have higher calcium, phosphorus, and calcium × phosphorus product values but similar CRP levels (mean 16.28 ± 11.28 mg/dL) when compared to low viscosity group. We suggest that long HD duration and high calcium and phosphorus levels leading endothelial dysfunction and microinflammation may cause hyperviscosity, thus increased thrombogenicity and atherosclerotic lesions.

Iron deficiency can increase the number of platelets in blood, which is linked with a hypercoagulable state [52]. As serum iron is an important regulator of thrombopoiesis and normal iron levels are required to prevent thrombocytosis by acting as an inhibitor [53], we selected patients with normal iron parameters.

Although anemia has been associated with increased rates of death and complications in patients with chronic or end stage kidney disease [54, 55] a reduced hematopoietic response to ESAs has also been associated with an increased risk of an adverse outcome [56–60]. It has been shown in the TREAT cohort that the patients with the poorest initial response to ESA treatment received the highest average doses of ESAs and had the highest event rates and the poor outcome [23]. Of note, a higher rate of increase in the hemoglobin level was not associated with greater risk. Indeed, patients with the greatest increase in hemoglobin level during the initial month of therapy had the lowest risk of clinical events [24]. In our study, higher dosage of rHuEPO was required for similar Hb levels in hyperviscosity group without iron deficiency. 

Higher ESA requirements may lead to an increased risk for adverse outcomes due to the underlying factors affecting rHuEPO response, such as inflammation, and the potential nonerythropoietic effects of greater administered ESA doses [23, 61, 62]. However, the pathway inducing inflammation-mediated EPO resistance has not been determined [63]. The inflammatory cytokines are in turn thought to directly inhibit erythropoiesis and promote apoptosis of erythroid precursors [64, 65]. In our study, the serum CRP levels of both groups with high and low viscosity were similar and slightly increased; the rHuEPO hyporesponsiveness in high viscosity group was not associated with clinically evident inflammation. We suggest microinflammation due to increased cytokine production as a result of comorbidity [such as heart failure, atherosclerosis, and volume overload], accumulation of advanced glycation end products, carbonyl stress, and oxidative stress could influence both viscosity and rHuEPO resistance. 

In this present study, the frequency of thromboembolic complications of arteriovenous fistula [AVF] was significantly higher in patients with hyperviscosity. Vascular access failure is a major contributor to the morbidity and mortality of hemodialysis patients [66, 67]. AVF prevention and management of its complications remain the safest and most comfortable solution to ensure AVF survival and thus a satisfying survival and quality of life in MHD patients [68]. Regression analyses of our study revealed that rHuEPO hyporesponsiveness was the major determinant of hyperviscosity; therefore we strongly suggest that higher EPO dosage need in MHD may predispose thromboembolic events of AVF. Hyperviscosity seems to play a key role for thromboembolic AVF complications of rHuEPO hyporesponsiveness. 

## 5. Conclusion

In conclusion, according to our findings we suggest that the hyperviscous state of the hemodialysis patients may arise mostly from the inflammatory situation of long-term HD, the calcium-phoshorus mineral abnormalities and rHuEPO hyporesponsiveness and related high dosage requirements. Hyperviscosity seems to play a critical role for the thrombotic side effects of rHuEPO and not hemoglobin levels but high serum viscosity level is probably associated with high serum calcium-phosphorus levels, endothelial dysfunction, and vascular calcification. Therapies targeting hyperviscosity may reduce cardiovascular complications of the hemodialysis patients. Further prospective studies with long-term followup are needed to show the exact mechanisms of hyperviscosity in hemodialysis patients. 

## Figures and Tables

**Table 1:** Demographic and biochemical characteristics of the whole study group.

	Study group (*n* = 84)
Age (years)	54.88 ± 13.7
Gender (F/M)	30/54
Mean plasma viscosity (mPas)	2.52 ± 0.65
MHD duration (months)	115.41 ± 81.72
Mean serum Hb level (g/dL)	11.28 ± 1.53
Mean serum WBC count (/*µ*L)	7.40 ± 1.84
Mean serum Plt count (/*µ*L)	215.89 ± 64.35
Median serum PTH level (pg/mL)	397.85 (498.30)
Mean serum Ca level (mg/dL)	9.31 ± 0.65
Mean serum P level (mg/dL)	4.95 ± 1.00
Mean Ca × P product	46.41 ± 10.80
Mean serum CRP level (mg/L)	16.28 ± 11.28
Mean serum albumin level (g/dL)	3.64 ± 0.35
Median serum ferritin level (ng/mL)	531.00 (331.50)
Total parenteral iron therapy (U/12 months)	20000 (3000)
Total rHuEPO usage (U/kg/week)	295.00 (422.35)
rHuEPO/Hb	25.49 ± 20.48

**Table 2:** Demographic and biochemical characteristics of the study groups.

	Group 1 (*n* = 42) (lower viscosity)	Group 2 (*n* = 42) (hyperviscosity)	*P* value
Age	55.4 ± 14.8	54.3 ± 12.5	0.728
HD duration (months)	97.5 ± 83.1	133.2 ± 77.1	0.044
PTH (pg/mL)	492.3 ± 500.3	562 ± 436	0.498
Calcium (Ca) (mg/dL)	9.1 ± 0.6	9.5 ± 0.6	0.009
Phosphor (P) (mg/dL)	4.7 ± 1	5.1 ± 0.8	0.035
Ca × P	43.4 ± 11.4	49.3 ± 9.3	0.011
Albumin (g/dL)	3.7 ± 0.3	3.5 ± 0.3	0.171
URR (%)	69.4 ± 5.8	71.7 ± 6.1	0.079
Hb (g/dL)	11.4 ± 1.7	11.1 ± 1.3	0.439
Platelet (/*µ*L)	221.8 ± 66.4	209.7 ± 62.3	0.396
CRP (mg/L)	16.7 ± 11.4	15.8 ± 11.2	0.735
Gender (F/M)	12/32	18/24	0.255
Need of rHuEPO usage for Hb 11-12 g/dL (%)	31	66.7	0.004
rHuEPO dosage (U/kg/week)	204.2 (350.8)	356.4 (295.7)	0.006
rHuEPO/Hb	20.39 ± 19.83	30.71 ± 20.04	0.021

**Table 3:** Thrombotic complications and mortality of the study groups.

	Group 1 (*n* = 42) (lower viscosity)	Group 2 (*n* = 42) (hyperviscosity)	*P* value
Thrombotic AVF complications (*n*, %)	13, 30.9%	23, 54.7%	0.023
Acute myocardial infarction (*n*, %)	8, 19%	13, 30.9%	0.208
Deep venous thrombosis (*n*, %)	2, 4.7%	0	0.152
Pulmonary thromboembolism (*n*, %)	0	1, 2.38%	0.314
Cerebrovascular disease (*n*, %)	2, 4.7%	1, 2.38%	0.557
Mortality (*n*, %)	3, 7.1%	5, 11.9%	0.457

**Table 4:** Demographic and biochemical characteristics of the study groups.

	Patients with no rHuEPO (*n* = 21)	Patients with low dose rHuEPO (*n* = 31)	Patients with high dose rHuEPO (*n* = 32)
Age (years)	54.3 ± 8.9	57.6 ± 17.4	53.5 ± 13.4
Mean plasma viscosity (mPas)	2.5 ± 0.6	2.4 ± 0.7	2.6 ± 0.5
MHD duration (months)	141.3 ± 87.6	83.7 ± 79.8	120.0 ± 75.5
Mean serum Hb level (g/dL)	13.1 ± 1.1	10.7 ± 1.06	10.5 ± 1.02
Mean serum Plt count (/*µ*L)	216.4 ± 60.4	209.7 ± 59.5	219.2 ± 70.1
Median serum PTH level (pg/mL)	409.5 ± 372.07	422.4 ± 359.8	649.2 ± 540.7
Mean serum Ca level (mg/dL)	9.3 ± 0.4	9.1 ± 0.7	9.4 ± 0.6
Mean serum P level (mg/dL)	4.8 ± 0.8	4.6 ± 1.03	5.1 ± 1.02
Mean serum CRP level	12.4 ± 11.6	18.08 ± 10.03	17.2 ± 11.5
Mean serum albumin level (g/dL)	3.7 ± 0.2	3.6 ± 0.3	3.6 ± 0.4
Total parenteral iron therapy (U/12 months)	2523.8 ± 1141.4	2231.3 ± 1079.2	2278.3 ± 1644.5
Mean URR (%)	68.5 ± 5.6	70.1 ± 6.6	71.9 ± 5.8
